# Pseudo-hypopion inverse avec polycorie congénitale

**DOI:** 10.11604/pamj.2014.19.108.3960

**Published:** 2014-09-30

**Authors:** Soufiane Berradi, Mounir Lezrek

**Affiliations:** 1 Université Mohammed V Souissi, Service d'Ophtalmologie A de l'Hôpital des Spécialités, Centre Hospitalier Universitaire, Rabat, Maroc

**Keywords:** Pseudo-hypopion, polycorie, congénitale, Pseudo-hypopion, polycoria, congenital

## Image en medicine

Patient de 34 ans, ayant une polycorie congénitale bilatérale (Flèches blanches), aphaque ODG (opéré à l’âge de 4 ans pour cataracte congénitale bilatérale sans implant), ayant bénéficié, suite à un décollement de rétine avec prolifération vitréo-rétinienne avancée et trou maculaire stade 4 de l'oeil droit d'une vitrectomie, pelage de membranes de PVR, ablation de la limitante interne, endolaser et tamponnement par huile de silicone. Les suites post-opératoires immediate étaient simples. Un an après l'intervention, le patient a consulté pour BAV progressive de l'oeil droit. L'examen au biomicroscope a objective un pseudo-hypopion inverse (Flèches noires) correspondant au passage de l'huile de silicone émulsifiée en chamber antérieure et au niveau de l'angle irido-cornéen, associé à une hypertonie oculaire à 26 mmHg. Devant ce tableau clinique, on a realisé l'ablation de l'huile de silicone à deux voies en 23 gauges. Le contrôle per-opératoire du segment postérieur a montré une rétine réappliquée et le tonus oculaire s'est rapidement normalisé. L'ablation d'huile de silicone représente donc le geste thérapeutique indispensable devant cette complication du tamponnement interne par huile de silicone.

**Figure 1:**
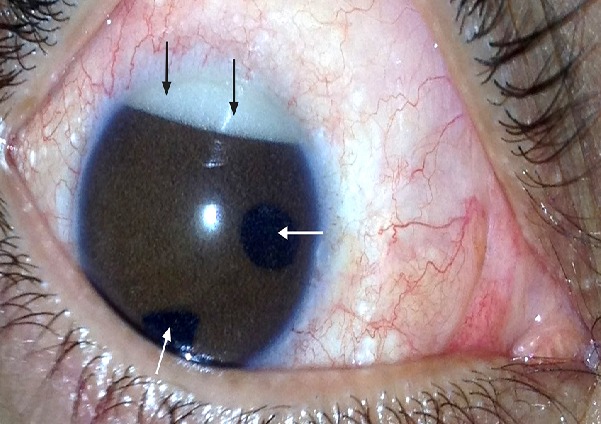
Pseudo-hypopion inverse de l’œil droit (Flèches noires) associé à une polycorie (Flèches blanches) chez un patient de 34 ans, aphaque (opéré à l'enfance pour cataracte congénitale)

